# Feedback About a Person’s Social Context - Personal Networks and Daily Social Interactions

**DOI:** 10.1007/s10488-023-01293-8

**Published:** 2023-08-24

**Authors:** Marie Stadel, Gert Stulp, Anna M. Langener, Timon Elmer, Marijtje A. J. van Duijn, Laura F. Bringmann

**Affiliations:** 1 Department of Sociology, University of Groningen, Groningen, The Netherlands; 2 Department of Psychometrics and Statistics, University of Groningen, Groningen, The Netherlands; 3 Inter-University Center for Social Science Theory and Methodology, University of Groningen, Groningen, the Netherlands; 4 Groningen Institute for Evolutionary Life Sciences, University of Groningen, Groningen, The Netherlands; 5 Department of Psychology, University of Zurich, Zurich, Switzerland

**Keywords:** Personalised psychotherapy, Experience sampling methodology (ESM), Egocentric networks, Social networks, Personalised feedback

## Abstract

The social context of a person, meaning their social relationships and daily social interactions, is an important factor for understanding their mental health. However, personalised feedback approaches to psychotherapy do not consider this factor sufficiently yet. Therefore, we developed an interactive feedback prototype focusing specifically on a person’s social relationships as captured with personal social networks (PSN) and daily social interactions as captured with experience sampling methodology (ESM). We describe the development of the prototype as well as two evaluation studies: Semi-structured interviews with students (N = 23) and a focus group discussion with five psychotherapy patients. Participants from both studies considered the prototype useful. The students considered participation in our study, which included social context assessment via PSN and ESM as well as a feedback session, insightful. However, it remains unclear how much insight the feedback procedure generated for the students beyond the insights they already gained from the assessments. The focus group patients indicated that in a clinical context, (social context) feedback may be especially useful to generate insight for the clinician and facilitate collaboration between patient and clinician. Furthermore, it became clear that the current feedback prototype requires explanations by a researcher or trained clinician and cannot function as a stand-alone intervention. As such, we discuss our feedback prototype as a starting point for future research and clinical implementation.

Measuring patients’ behaviours, cognitions, affect and other symptoms in daily life settings has been the focus of much recent methodological and substantive research (Ebner-Priemer & Trull, 2009). Specifically, Experience Sampling Methodology (ESM), also termed Ecological Momentary Assessment (EMA), offers a more fine-grained view of psychopathology compared to traditional assessments in a lab or a cross-sectional survey (Myin-Germeys & Kuppens, 2021). By repeatedly measuring symptoms via brief questionnaires in different daily situations, ESM allows for examining the context-specificity of symptoms, such as levels of depressed mood in situations when a patient is alone versus in the company of friends. Such information is considered especially useful by clinicians and clinical researchers (Piot et al., 2022). Contextual information itself – such as how much time patients spend alone versus how often they interact with others and what quality these social interactions have – can also be insightful for psychotherapy. Thus, ESM may not only be useful for assessing symptoms but also for capturing the daily situations or contexts a patient is in.

Until now clinical ESM research has primarily focused on measuring symptoms such as affect and cognitions, which are assumed to be at the core of psychopathological complaints (Wichers et al., 2021). Particularly personalized feedback based on such symptom data has offered valuable insights into the patient’s complaints during daily life and is increasingly used as a tool in psychotherapy (Bringmann et al., 2021; Bos et al., 2022). Incorporating also the context of symptoms into personalized feedback offers insight into different situations in the patient’s daily life and enables discussions about specific moments similar to self-monitoring records commonly used in cognitive behavioural therapy (CBT, Beck and Beck, 2011). These discussions may involve the patient’s daily activities, specific people the patient spends time with, and the intensity of symptoms experienced. This approach, as exemplified by von Klipstein and colleagues (2023), can reveal intervention targets, such as a certain social environment the patient finds particularly pleasant. By intervening on a contextual factor (e.g., encouraging the patient to seek out the pleasant social environment more frequently), mood could improve. Thus, instead of looking at mood or symptom levels in isolation, capturing the context in which a symptom is experienced more or less intensely can have therapeutic value. Thus, there is a pressing need for clinical researchers to also examine individual contextual factors and their relation to mental health (Kinderman et al., 2020).

One particularly important factor is the social context of a patient which influences psychopathology and may contain potential intervention targets. *Social context* refers to the daily social life – *social relationships *and *social* *interactions* – of an individual. Social context can improve and aggravate mental conditions. For instance, the perception and reception of social support act as a buffer from stressors, thereby protecting people against developing a mental disorder (Southwick et al., 2016). Social support also helps in dealing with, and recovering from existing psychopathology (Brown et al., 2011). In contrast, adverse social interactions or the absence of social contact can have deteriorating effects on mental health (Bertera, 2005; Rook, 1984; Lakey & Cronin, 2008; Yanos et al., 2001). Additionally, several diagnoses include interpersonal problems which are increasingly investigated as a transdiagnostic factor involved in psychopathology (McEvoy et al., 2013, Girard et al., 2017).

Daily *social interactions*, affect, and other symptoms (e.g., maladaptive cognitions, fatigue or concentration difficulties) can be captured well with diary methods such as ESM (Brown et al., 2011; Reis & Wheeler, 1991). In existing ESM research, however, assessment is often limited to asking whether someone was in the company of others, how the company was perceived, and which social role the interaction partner had (e.g., friend or family member; for a review see Langener et al., 2023). Such assessments ignore the effects of *social relationships* with specific interaction partners (e.g., the younger sister versus the older brother).

Participants have a particular relationship with a specific interaction partner that is defined by the history of their social interactions. Such social relationships are more stable than daily interactions and can be captured through a personal social network (PSN; Perry et al., 2018). To collect a PSN, usually participants need to provide a list of significant social contacts, such as those they are close to or have interacted with over the past year. Once these contacts are identified, their relationship with the respondent and further characteristics (e.g., age and gender) can be assessed. A PSN can illustrate the social support resources of a patient, highlight potentially problematic relationships (e.g., in the case of substance abuse disorders; Stone et al., 2016) and serve as a basis for interpersonal interventions. Such clinical application of social networks has shown to be useful in treating patients with severe mental illness (Nicaise et al., 2022).

Combining information from PSN (i.e., the social relationships of the patient) and ESM assessments (i.e., symptoms and social interactions in daily life) provides detailed insights into a patient’s social context (Sun, Harris & Vazire, 2020) which may be helpful for treatment. The patient and therapist could for example learn whether there are social network members (e.g., an old friend from high school) that the patient has a good relationship with, but in daily life never reaches out to. Additionally, a combination of ESM and PSN allows seeing who in the patient’s environment is particularly supportive and can be reached out to in a crisis (e.g., the brother living close by).

However, the research on how to transform information gathered by PSN and/or ESM into relevant feedback on a person’s social context that is helpful for clinical practice, is still in its infancy. Existing feedback tools, such as the ESMvis (Bringmann et al., 2021), PETRA (Bos et al., 2022) or Therap-i tool (von Klipstein et al., 2023) do not include information on social relationships and social interactions with specific interaction partners yet, since capturing such information requires new assessment methods combining ESM and PSN. For developing effective feedback tools that do include this information, we need to understand how to summarise and present social context information to patients.

In this article, we thus present a series of studies in which we develop and evaluate a social context feedback prototype, which is informed by data from ESM and personal network assessments. Our feedback prototype visualizes various aspects of a person’s social context and its two components – social interactions and social relationships. First, we describe the development of this feedback prototype (Part 1). Subsequently, we present the results from a qualitative evaluation of the prototype via semi-structured interviews with student participants (Part 2) and a clinical focus group (Part 3).

## Part 1: Building an Interactive Social Context Feedback Prototype

We conducted a feasibility study, exploring the combination of PSN and ESM to capture the social context of university students. Based on the collected personal network and ESM data, we aimed to generate a feedback prototype that may be particularly relevant for applications in clinical practice. In the remainder of this section, we first describe the collected data, before discussing our strategy in developing the feedback prototype. Finally, we describe the features of the developed feedback prototype. The assessment instruments, detailed data collection descriptions and supplementary materials for all studies described in this paper can be found on our Open Science Framework project page.

### Methods

#### Participants & Procedure

Our study procedures and data management followed ethical standards and legal requirements laid out by the GDPR. Our research was approved by the Ethical Committee of the Faculty of Behavioural and Social Sciences at the University of Groningen.

In October 2021^1^, we recruited (N = 23) undergraduate students from the university’s participant pool who possessed an Android smartphone to use during the study period. Participants received either financial reimbursement or study credits for taking part in the study. Students who signed-up were immediately directed to a baseline questionnaire. Subsequently, all participants were divided into two groups that were composed as similarly as possible. Based on self-reported social behaviour in the baseline questionnaire, we composed each group to consist of both very socially active (i.e., interacting multiple times daily) and minimally socially active (i.e., interacting a few times per week) individuals. Then we invited each group to an instruction session. Group 1 (N = 11) completed a personal network assessment before and after an ESM period, Group 2 (N = 12) completed only the second network assessment (for more information we refer to our assessment materials on OSF).

#### PSN Assessments

We adapted open-source code from the GENSI tool (Stark & Krosnick, 2017; Stulp, 2021) and implemented it in the ESM software m-Path (Mestdagh et al., 2022). To obtain a comprehensive list of names of our participant’s social contacts we used three name generating questions: First, participants were prompted to come up with names of people they interact with during their daily life. Second, we asked for names of individuals that they have less frequent contact with, but who are still important contacts that they could reach out to if needed. Third, we let participants check the listed names and asked them to add anyone who they felt belonged in their social network. Participants were encouraged to use their phones or any other resource that would help them come up with names. Subsequently, we asked about the characteristics of each network member such as basic demographics (gender, age) and the relationship between the network members and the participant (e.g., closeness, frequency of online and in-person contact, and relationship type). Lastly, we assessed connections between network members by asking which network members have (any form of) contact which each other.

Participants went through a slightly modified version of the same procedure after the ESM period. All ratings from the pre-ESM network (of Group 1) and names of all encountered interaction partners during the ESM period (see below) were saved and displayed on the screen. First, participants could remove individuals from the network. Then, participants were presented with the three name generators which this time asked them to only add names that were not already present. Subsequently, participants were asked to reconsider and if needed adjust all ratings of each network member and answer all questions for the newly added members.

#### ESM Assessments

***Schedule.*** The assessment period lasted 28 days. Participants would receive push notifications, prompting *signal-contingent* assessment moments. The schedule consisted of a combination of 2 semi-fixed assessment moments (morning and evening questionnaires) and 4 semi-random measurement prompts (daily questionnaires). To capture social interactions, participants were instructed to log every face-to-face, video or phone call interaction they had that lasted longer than 5 min by clicking a button in the m-Path app (i.e., *event-contingent* reporting). Participants received an email each week containing their compliance rate as well as the number of times they logged interactions. In case no interactions were logged we asked participants whether they experienced any technical difficulties. More details regarding the ESM assessment schedule can be found on OSF.

**Content.** The signal-contingent questionnaires assessed momentary affect, activities since the last assessment, whether the participant is currently alone and how being alone/in company is perceived.

The social interaction questionnaire assessed momentary affect and detailed information about the social interaction that took place (interaction mode, timing, duration, location, type, content and the participant’s perception of the interaction). Most importantly, we assessed the interaction partner(s) of the social interaction. For this, the participant was presented with the list of names from the personal network assessment. If a new interaction partner was encountered, this name could be added to the list and was saved for future assessments.

#### Feedback Development Strategy

**Intended Use.** We built on an existing feedback format for psychotherapy patients (Therap-i; Riese et al., 2021; von Klipstein et al., 2023), and extended it with regards to *social contex*t. The Therap-i feedback tool and our prototype are intended to be used by a (trained) therapist and patient together during regular outpatient psychotherapy. Therapist and patient set up an ESM diary which assesses variables relevant to their treatment plan. Then, the patient completes ESM – and for our prototype also PSN – assessments over the course of a few weeks. Based on this data, an interactive digital feedback report is generated. This report contains different figures that allow one to inspect the captured variables over the data collection period and identify patterns (e.g., associations between social interaction and mood). This feedback report is explored and discussed together during therapy sessions. Given that such tools are still in the development and early implementation phase, the set-up of the personalized assessments, the generation of the feedback, and the identification of discussion points need to be supported by a researcher.

**Content.** In our feedback report, we aimed to give participants an overview of their social relationships (PSN data) as well as their social interactions and how these relate to mood (ESM data). We included mood as a placeholder for any symptom a clinician and patient may be interested in. Specifically, we wanted our feedback to answer a number of relevant clinical questions (see Table 1). These questions were selected via discussions in the research team and cover aspects of social context relevant to different psychotherapeutic approaches such as CBT, (Beck & Beck, 2011), Interpersonal Psychotherapy (IPT, Weissman et al., 2017) or Cognitive Behavioural Analysis System of Psychotherapy (CBASP, McCullough, 2003).

**Development Procedure**. In order to develop our feedback visualizations, we selected a participant from Group 1 (i.e., one who completed both, pre- and post-ESM network assessments) who came closest to the overall median number of social contacts^2^ (N = 42). Based on the data from this participant, feedback graphs were explored and a prototype for a report was generated. This prototype was then also tested for its functionality on the participant with the smallest (N = 10) and largest number (N = 80) of network members.

### Results: The Feedback Prototype

**Fig. 1:**
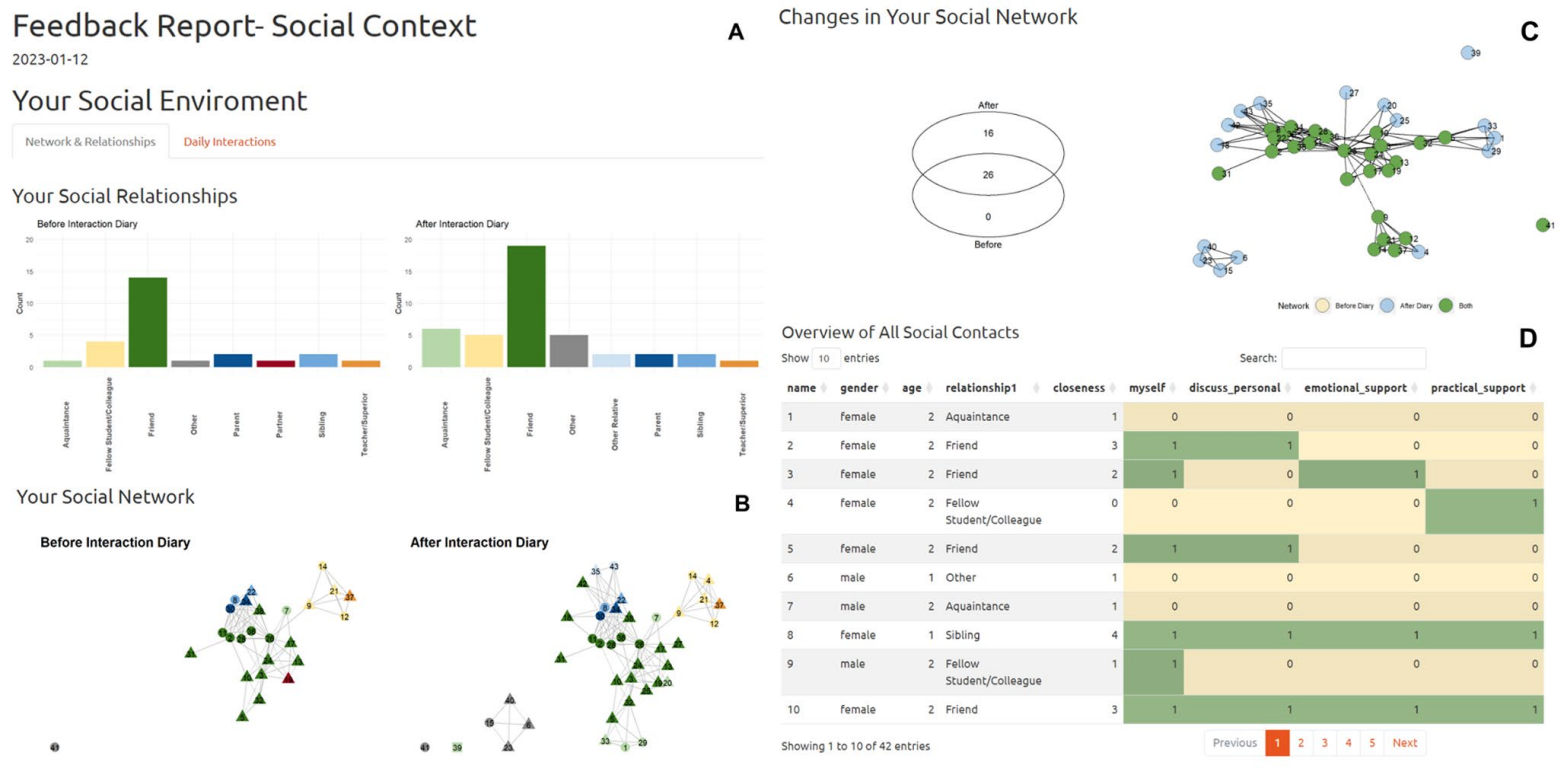
Social Context Feedback Prototype ‘Network & Relationships’ *Note*. In order to safeguard the anonymity of our participants some information in the graphs depicted in this paper was altered or hidden (e.g., names of interaction partners; exact dates and times of interactions).

**Fig. 2:**
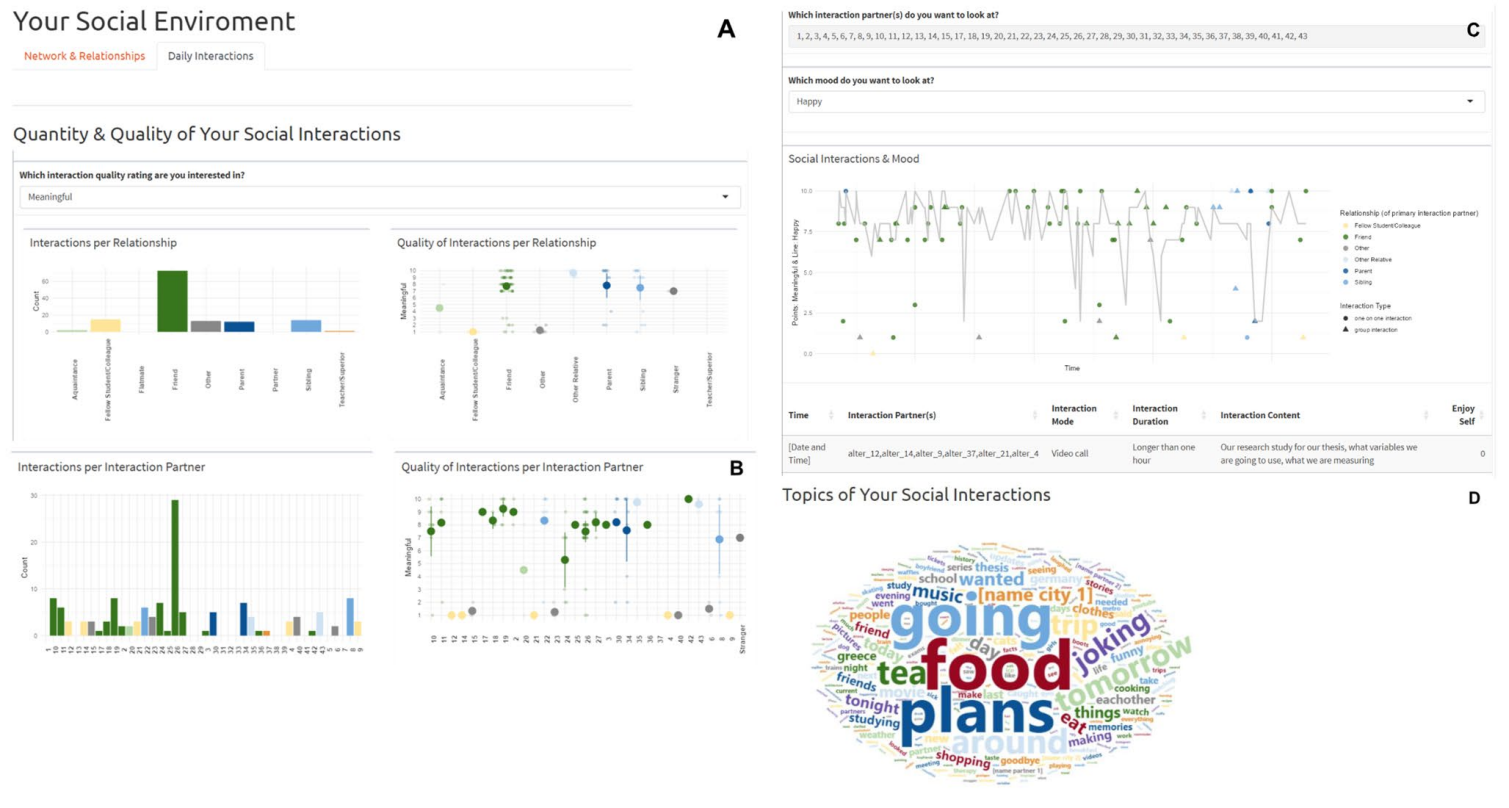
Social Context Feedback Prototype Tab ‘Daily Interactions’ *Note*. The bars in the interaction quality graphs depicted on the right of panels A and B indicate 95% confidence intervals around the mean, which is represented by the thick dot. In order to safeguard the anonymity of our participants some information in the graphs depicted in this paper was altered or hidden (e.g., names of interaction partners; exact dates and times of interactions).

We developed an interactive social context feedback report prototype using RMarkdown (Xie et al., 2021) and Shiny (Chang et al., 2021) which consists of two parts: One detailing the network and social relationships of a participant (Fig. 1) and one summarising and visualising social interactions and their relations with other variables captured during ESM assessments (Fig. 2). In the last column of Table 1, we indicate which figure can answer which specific clinical questions about the patient’s social context.

**Table 1:** Clinically relevant questions guiding the feedback development

	Clinically relevant question	Feedback report section
PSN	How many network members does the participant have?	Figure 1 A & 1 D
	Does the network change over the course of the assessment period or intervention?	Fig. 1B & 1C
	Does the participant have (emotional/practical) support resources?	Figure 1D
	How does the participant perceive social relationships?	Figure 1D
ESM	How socially active is the participant in daily life?	Figure 2 A & 2B
	Of what quality are the social interactions of the participant?	Figure 2 A; 2B & 2D
	To what extent are social interactions related to mood or other symptoms?	Figure 2 C
	Are there particularly impactful social interactions? If so, what happened during these?	Figure 2 C
PSN + ESM	Does the participant use available social resources in daily life?	Figure 2 A & 2 B
	With which network members does the participant interact?	Figure 2 B
	Social interactions with which network members are perceived particularly positively or negatively?	Figure 2B & 2 C
	Does the participant’s overall perception of a relationship match what is happening in daily life?	Figure 1D vs. Figure 2 A & 2B

#### Network & Relationships

The ‘Network & Relationships’ tab contains four sections: First, we show two bar graphs visualizing how many people of a certain social role are present in the participant’s network before and after the ESM period (labelled in the figures as ‘interaction diary’). In Fig. 1A one can see, that this participant’s network consists mostly of friends. Additionally, before the ESM period this person had a romantic partner and after the ESM period there was no longer a romantic partner; we thus have captured a break-up during our study period.

Second, we plot the complete personal network of the participant before and after the ESM period (Fig. 1B). Thereby, we visualize the connections between the different network members (i.e., who has contact with whom) as well as the name^3^ and social role of each network member.^4^ Network member 41, for instance, is not reported to know any of the other network members, while the family members (i.e., the blue nodes) of the participant are a closely connected group. In this plot, we can also see that the former romantic partner (network member 19) now is categorized as a friend.

Third, we specifically visualize the changes in people included in the network. We indicate with colour, which network members were included in both network assessments, and which were only included in the first or the second assessment (i.e., which network members were actively removed during the post-network assessment^5^ or which members were newly added either during ESM or the post-network assessments, respectively; Fig. 1C). For this person, no one from the initial network assessment was deleted, but 16 new network members were added. For example, the group including numbers 6, 15, 23 and 40 were added during or after the diary.

Last, the ‘Network & Relationships’ part also contains a table of all network members, including demographic characteristics (i.e., gender, age), social role and dichotomous relationship ratings (e.g., whether the participant received emotional or practical support from the person; Fig. 1D). In this case, network member 10, a female friend in her 20s, appears to be quite close to the participant, providing emotional and practical support. The participant indicated being able to be themselves with this person and discusses personal issues with her. The same applies to the participant’s sister (number 8) who is under 20 years old. Other relationships, with fellow students/colleagues or with acquaintances, are rated as less close.

#### Daily Interactions

The ‘Daily Interactions’ tab contains three sections that concern the data collected during the ESM period. The first section shows the quality and quantity of daily social interactions of a participant (Fig. 2A & B). This section contains in total four figures in a two-by-two grid. The left two figures visualize the number of social interactions, specifically how many interactions the participant had per social role (Fig. 2A) and per specific interaction partner/social network member (Fig. 2B). The overview per specific interaction partner clearly illustrates the differences in interaction quantity between interaction partners of one social role category (e.g., friend number 26 versus number 42), highlighting the value of our detailed assessment approach. This participant interacted most frequently with friends – particularly number 26. The two right graphs visualize the quality of social interactions, again split by social role (Fig. 2A) or by specific partner (Fig. 2B). Here, the full raw data is visualized, showing not only average quality (big dot) but also the variation in quality across all interactions. The two quality graphs change interactively, depending on which quality indicator is selected via the dropdown menu above this section. In the example in figure 2 we show as how meaningful the participant rated the interactions with the different interaction partners – most interactions with friends are rated 7 out of 10 or higher. Only interactions with one friend (number 24), were rated less meaningful on average. The participant also did not seem to consider the (few) social interactions with fellow students/colleagues as meaningful. This matches how the participant rated the quality of the relationships with fellow students (see part *Network & Relationships*).

The second section contains a timeline graph which displays affect and social interactions across the study period (Fig. 2C). Again, the graph changes interactively depending on which interaction quality indicator and affect variable are selected in the dropdown menus. In the example, we show as how meaningful a participant rated an interaction (dots) and the participant’s level of happiness (grey line). The participant was mostly happy, with only a few moments when happiness was temporarily rated low. One of these moments occurred towards the end of the ESM period, when more interactions with family members (blue dots) were reported. When examining the content descriptions of these interaction moments, it becomes clear that the participant visited family abroad for a few days and was sad to leave.

We can see such details about specific social interactions by clicking on the respective data point. At the bottom of the graph, more information about the selected interaction is then displayed. For our example participant, we were curious to find out why the participant did not enjoy interacting with fellow students and learned that all these interactions were about the thesis project – and that this project was not progressing very well. For the timeline graph, there is the additional option of only displaying interactions with specific interaction partners (e.g., we could choose to only select number 19 – the former partner, now friend). Then interactions with these partners can be explored in more detail.

In the last section of this part, we show a word cloud with the most frequent terms participants used in qualitative descriptions of their social interactions (Fig. 2D). We removed common stop words as well as verbs referring to the activity of ‘talking’. Our example participant’s interaction descriptions most often mentioned ‘food’, ‘plans’ and ‘going [somewhere]’, but besides that, there was a range of other topics (e.g., ‘music’ or different places).

## Part 2: Evaluating the Prototype – Student Sample Interviews

In 2022, we collected a second dataset from another student sample^6^. After the main assessment period ended in which personal networks and social interactions were captured, these students received feedback about their social life during the study. We generated a personalized feedback report according to our prototype presented above for each participant and discussed its content with them in a one-on-one session. Directly after this feedback discussion, students were interviewed about the feedback session in order to evaluate the feedback prototype. In the following, we will describe this evaluation study in detail.

### Methods

#### Participants & Procedure

We recruited (N = 19) student participants from the university’s participant pool. The study procedure was almost identical to the data collection described in Part 1 with one addition: We conducted feedback sessions and an in-depth semi-structured interview to evaluate our study with each participant one week after the other assessments were completed.

There were a few small modifications to the ESM and PSN assessments to decrease participant burden (e.g., 5 signal-contingent prompts per day instead of 6). Furthermore, we used this data collection to compare signal-and event-contingent reporting of social interactions using a within-subject design. Thus, participants reported interaction for two weeks in an event-contingent design and two weeks in a signal-contingent design.

#### Feedback Session & Evaluation Interview

The feedback session/evaluation interview was conducted in a semi-structured format by two different researchers, each being responsible for half of the participants. At the beginning of the feedback session, we asked participants what kind of insights they obtained from the study just by completing the assessments and what kind of feedback regarding their social life (network and social interactions) they would be interested in. Then we walked them through their individual feedback report, explaining how to interpret the visualizations and what patterns can be seen based on their data. The goal of this session was not to evaluate the social life of the participants, but to test the prototype that we made. Thus, we refrained from giving advice to participants.

Following the walk-through of the report, we asked the participants about their impressions of the prototype. Specifically, we were interested in whether the feedback was (1) useful/insightful for them, and (2) understandable/complete. We also asked what they have taken away from the feedback and how this made them feel.

Lastly, we asked for concrete improvement suggestions and three quantitative ratings of the feedback tool with regard to understandability, insightfulness, and representativeness (1 = not at all to 10 = very much).

The two researchers who interviewed the participants took detailed notes which were later analysed by one of the researchers. The qualitative analysis consisted of identifying themes and counting the number of participants mentioning each theme.

### Results

#### Did Students Gain New Insight Into Their Social Life From the Feedback?

**Fig. 3:**
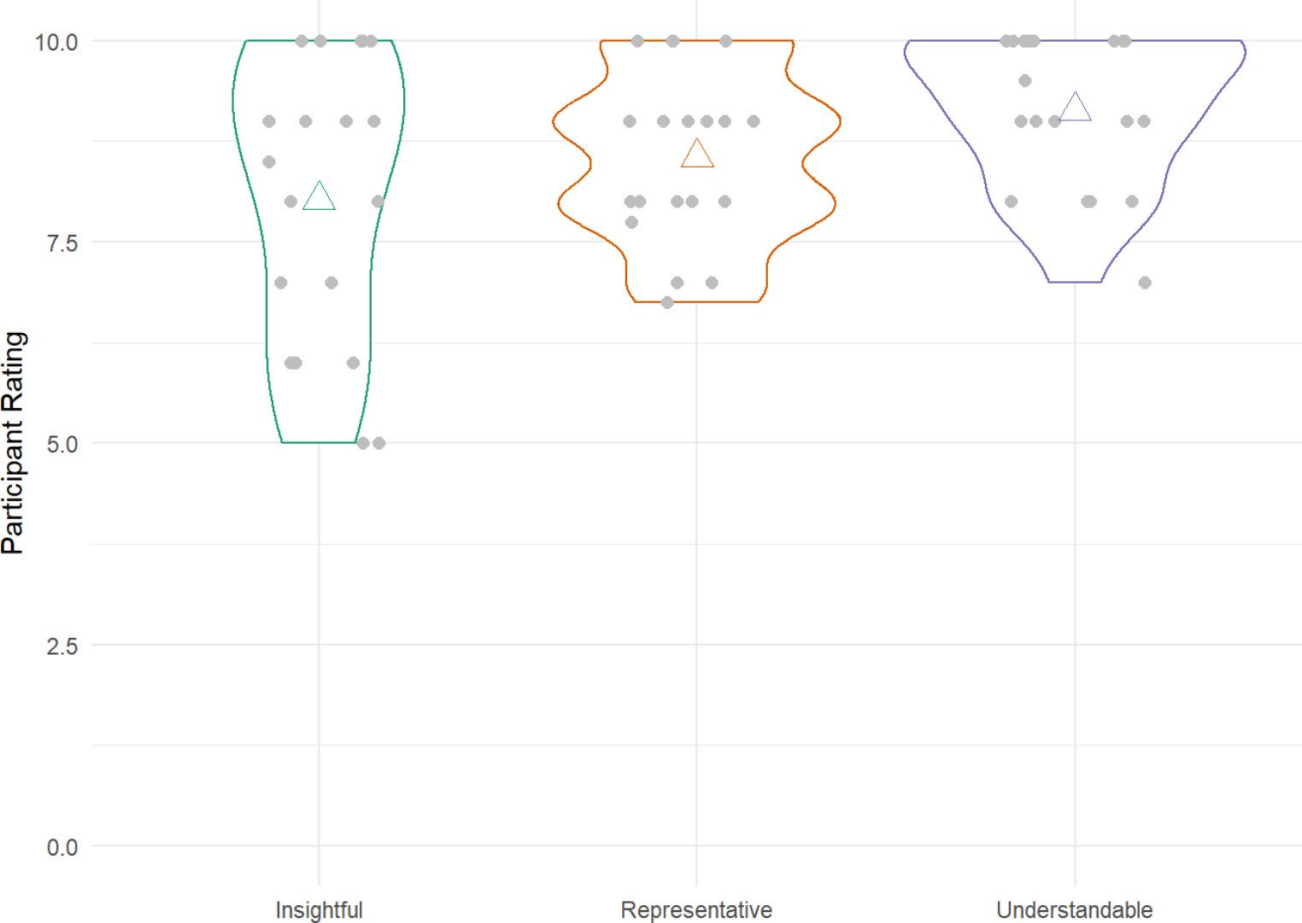
Quantitative ratings of the feedback report *Note*. The triangles indicate the mean rating, each grey dot is a rating by a single participant, the shape of the line shows the (density-)distribution.

Based on the quantitative ratings, students overall considered the feedback insightful (median = 8.5), but how insightful exactly varied (min = 5, max = 10; see Fig. 3)^7^.

When asked what exactly participants took away from the feedback graphs, four participants (21%) noted not having learned anything new and six participants (32%) mentioned that they liked seeing the feedback as a summary of their social life, but that also for them the specific details were topics they were already aware of.

Completing the assessments rather than receiving feedback seemed to create most awareness. Most participants (N = 16; 85%) noted that the whole study procedure made them really aware of their social life and that they liked the opportunities to reflect. Multiple people (N = 4, 22%) specifically indicated having gained insights into how their mood and social interactions are connected (e.g., feeling lonely on days with few interactions) while completing the ESM assessments. Participants also reported noticing different types of social interactions (e.g., actively doing something together vs. just meeting to chat) and that social interactions with different people had different effects on them. The study helped them realize which social contacts had positive or negative effects. Two participants (11%) reported that they made changes to their social life based on the assessment (i.e., decrease contact with a person that frequently costs them energy).

Participants noted that they appreciated the combination of PSN and ESM, as the network was a snapshot of their own view of their social life. The ESM period helped them evaluate this snapshot and in the second network assessment, they could adjust their perception. Most participants (N = 16; 85%) felt more connected to the people around them and understood themselves better after the assessment period.

The feedback did still create a few new insights: Six participants (32%) indicated they learned more about the structure and stability of their social network. Participants further considered it insightful to learn about the q﻿uality of their relationships (N = 3) and social interactions (N = 11). Three participants (16%) noted they learned something from the feedback about the relationship between social interactions and their mood.

When inquiring how our feedback made participants feel, one participant noted that receiving feedback on negative relationships and social interactions can feel confronting. Four participants felt neutral about the feedback procedure and one participant indicated surprise that some topics did not match his perception. He explained that this is due to not carefully filling in the assessments. The remaining participants (N = 14) indicate a positive feeling, many noting the confirmation of their self-perception.

#### Was the Feedback Report Understandable and Complete?

Participants considered the feedback easy to understand (median = 9, min = 7, max = 10) and representative (median = 9, min = 6.75, max = 10) of their social life (see Fig. 3). Most people indicated having no trouble understanding the report in its entirety (N = 17), though a few participants noted that it would be hard without the researcher’s explanation (N = 5) or statistical education (N = 2). Three participants noted difficulties with understanding the social network visualizations. This seemed to be the case especially for large social networks.

The feedback provided mostly matched the expectations that participants had prior to seeing the graphs: Participants were interested in the number of social interactions they had (N = 2), with whom they interacted the most (N = 2), and what kinds of interactions they had with which network member (N = 2). Participants were particularly interested in their mood and how it is influenced by the quantity and quality of social interaction in general (N = 4), social interactions with specific people (N = 2), and different events and activities (N = 3). One participant noted to be specifically interested in understanding how to improve mood. Further, one participant wanted to know how the ratings of their relationship changed between the two network assessments and another participant wanted to know how their friend groups are connected.

### Improvement Suggestions and Additions Named by the Students

When asking for improvement suggestions, seven participants had none. Also, regarding additions to the report, most participants (N = 12) did not miss anything. Five participants noted explicitly that they liked the use of colour and four participants complimented the word cloud. A few participants made specific suggestions for improvements and additions (see Table 2).

**Table 2:** Participant suggestions for improvements of the graphs and additions to the report

Suggestion	N
Provide an option to zoom into (parts of) the social networks	1
Show a clear distinction between online/offline interactions	1
Show how many social interactions fall under a specific communication episode type (e.g., gossip, catching up)	2
Compare moments alone versus moments in company	1
Allow zooming in on the closest friends	1
Show cumulative frequencies of social interactions	1
Show the relationship between conversation topic and mood	1
Allow for the examination of specific timeframes (e.g., a day, a week)	1
Provide more insight into recurring patterns	1
Show the duration that you spend with a specific person (not just the count of interactions)	1
Add a word cloud for interaction partners	1
Minor adjustment to the graphs (e.g., change colour or adjust axis labels)	8

## Part 3: Evaluating the Prototype – Clinical Focus Group

After the development of the feedback prototype and the first test with student participants, we wanted to also receive input from a clinical population as the tool is mostly intended to aid patients and clinicians to obtain a detailed picture of a patient’s social life. Therefore, we organized a patient focus group.

### Methods

#### Participants

With the help of researchers at a mental health institution in the northern Netherlands, we recruited a group of five (former) mental health care patients by contacting participants from an ongoing study, who indicated they were open to being approached for follow-up research. During the ongoing study they participated in, these patients’ social context was captured via a PSN and ESM assessments which made them ideal candidates for the current focus group as they experienced the assessment methods and had an understanding of the collected data. Note that they did, however, not receive feedback graphs summarizing their own data during the focus group, but were shown an anonymised example from the student sample.

The resulting group consisted of four men aged between 30 and 70 and one woman in her early twenties. Most of the participants completed higher education. Each of them had experience with receiving psychotherapy – albeit for different disorders, namely depression, bipolar disorder, PTSD, and functional neurological disorder. Three participants were still in psychotherapeutic treatment at the time of the focus group discussion.

#### Procedure

The procedures of the focus group were approved by the institutional review board of the University Medical Centre Groningen (UMCG). Participants provided written consent for their participation and the publication of the focus group results. The discussions took 2 hours and participants were compensated for their time (20€) and travel costs. The discussion was held in Dutch, as all participants were native speakers. During the discussion, we asked participants to reflect on their experience with the measurement of their social context and what kind of feedback they would like to receive on the collected data. Thereby, we focussed specifically on what kind of feedback would be useful for psychotherapy. Additionally, we showed simplified and slightly improved parts of the feedback prototype, specifically Fig. 1A and B as well as Fig. 2A, B and C, which participants were asked to evaluate. At the end of the discussion, participants completed a short questionnaire assessing demographics and information about their current or past diagnoses and psychotherapeutic treatment. The discussion was audio-taped and summarized. The summary was sent to participants for approval.

### Results

After listening to the audiotapes and compiling a summary of the focus group discussion we conducted a thematic analysis. We identified five overarching themes that were discussed: (1) how representative the feedback is of the participants’ social life, (2) how the feedback could be useful for psychotherapy, (3) the privacy and autonomy of the participants in relation to the collected data, (4) norms and comparisons based on social context data, and (5) concrete improvements for our feedback prototype. Most themes were mentioned during multiple phases of the discussion (e.g., when asking for expectations for feedback and also when evaluating specific graphs). In the following, we provide a short description of the main points raised per theme.

#### Representativeness of the Feedback

Three participants appreciated that their whole life – including negative and positive moments – is covered in assessment and feedback. One participant, however, noted that it is sometimes difficult to summarise the content of social interactions and that the collected data (and displayed feedback) is naturally limited: *“Practice is more dynamic than the possibilities offered by the interface. I chose the highlights.“.*

#### Use for Psychotherapy

Participants differed in how useful they expected feedback on their own data to be: While three believed feedback can provide insight and create awareness for themselves, one participant said: *“My psychiatrist can do more with it than I can.“* Another participant picked up on this and indicated that a summary of daily life data can indeed be very useful for the therapist to learn more about the patient outside of sessions. It can be useful at the beginning of a treatment process – to get to know the patient – and throughout the treatment.

Participants agreed that the patient and therapist should examine the data collaboratively.

While one participant mentioned that therapists should be trained for using these data collection and feedback methods, the group did not expect therapists to in-depth analyse data before their joint session. Two of the participants mentioned that they would be willing to prepare a session with their own data, by pre-selecting topics or moments they would like to discuss. One of them explained that the graphs in the feedback report would be useful to help remember situations that are important to discuss with the therapist. In particular, it may help to remember not only impactful or negative interactions but also positive and less salient moments.

A strategy that seemed to be liked by the group is that the therapist and patient together examine moments with extreme responses (positive and negative) and then zoom in on those moments. Often, useful information can be uncovered by exploring specific social interactions in detail: What was discussed, and with whom? It was also considered relevant to identify relationships between social interactions and specific symptoms (e.g., mood). It seemed crucial for participants that symptoms relevant to the patient at hand are assessed, as opposed to a standard set of items. Further, participants stressed that an important part of their treatment is to build and improve coping skills. The discussion of coping skills in relation to specific daily life situations seemed very relevant to the group.

One participant was also interested in labelling particular positive and negative time periods (as opposed to moments) and being able to zoom in on these.

The prevailing conclusion raised by participants is that information about social relationships and interactions in daily life is valuable for treatment. One participant noted that data on social interactions will clearly show differences with regard to social interactions between depressive and non-depressive phases: *“If I’m in a depressive phase and then I’m presented with a diary like this, you can throw social interactions overboard – there are hardly any.“* The group, however, agreed that the feedback graphs need to be tested in practice (i.e., with a particular patient case) to determine their concrete use.

#### Data Privacy and Participant Autonomy

All patients indicated that they would appreciate using the real names of their social contacts instead of initials or nicknames for anonymisation purposes. This facilitates recognition and easier communication during the feedback process.

One participant cautioned that some people may not be comfortable with answering completely and honestly, if all details are shared with their therapist. The other participants actually considered it useful to make use of the therapist’s expertise and would be happy to share everything for that purpose.

A compromise that was discussed, is that a patient can decide whether or not a (specific) therapist is allowed to see all details or just part of them. Participants felt like the content of specific social interactions is particularly personal and participants would like the autonomy in clicking on the interaction moments they want to show. The information about specific interaction partners (e.g., Lisa versus Tom) was perceived as very personal as well, but again almost all participants found it useful to share this information with their primary therapist. For other mental health care professionals involved in a patient’s case, a more anonymised version would be preferred.

#### Norms and Comparisons

One participant wanted to know more about their own data in relation to general norms (e.g., is the way of handling social interaction deviant or similar to everyone else?). Other participants found such a comparison with others not desirable. At several moments of the discussion, a comparison with oneself in different time periods was brought up. This appeared to be favoured over comparisons to others by most participants.

#### Feedback Prototype Improvements Suggestions

The graphs were understood fairly quickly when presented to the participants – but they clearly did require an explanation from the researchers (or trained therapists). Participants considered all the displayed information interesting and would like to have these kinds of graphs of their own data. It was, however, questioned whether the information in the timeline graph can be displayed in a way that is easier to digest. One option to facilitate easier comprehension could be to start with a simpler graph that shows only the line indicating mood (or other symptoms) and the points representing social interactions. The additional dimensions of the social role of the interaction partner and group vs. one-on-one interaction were perceived as too much information at once. Based on a simpler line graph version, one could zoom into specific periods or moments and then see more detailed information about one moment.

## Discussion

We developed and illustrated an interactive feedback prototype providing detailed insights into a person’s social context – their social relationships and daily social interactions. We explored the use of this feedback prototype in two qualitative studies; semi-structured interviews with student participants and a focus group with (former) psychotherapy patients.

The feedback was perceived as interesting and useful by both the student participants as well as the patients in the focus group. They particularly liked seeing the relationship between social interactions and mood as well as the quality of specific social relationships and interactions. Various suggestions were made that will help to improve the feedback prototype, such as making the social network visualisation more interactive. The focus group provided patients’ ideas and expectations about using feedback in psychotherapy. It also highlighted a few issues to be cautious about such as privacy, the autonomy of participants over their data and the use of comparisons to participant’s own data during other time periods or the data of others.

In line with prior studies, our results suggest that insight and self-awareness are already gained from completing the assessments themselves even without the feedback (Bakker & Rickhard, 2018; Bos et al., 2020). Thus, our assessment seemed to pick up on important aspects of our participants’ (social) lives. Similar to the findings by Bos and colleagues (2020), participants described the feedback report as a confirmation of their self-perception. This does not mean that the feedback is not needed for clinical applications. A randomised controlled trial by Kramer and colleagues (2014) suggests that feedback has an added benefit beyond assessments. Moreover, the participants from our focus group considered the feedback a valuable summary of their social life for their therapist that can support their treatment. Thus, the feedback may aid communication and collaboration between therapist and patient (Piot et al., 2022).

Leertouwer and colleagues (2022a) found that participants do adjust their self-perception regarding their own affective experiences based on personalised feedback, even if the feedback is incorrect (Leertouwer et al., 2022b), we therefore need to be cautious about what kind of feedback we provide to patients and how. Our study further showed that participants may even already change their behaviour based on insights from the assessments, with two student participants mentioning that they made adjustments to their social life after the assessment phase of our study (e.g., seeing a person with whom interactions were of low quality less often). On the one hand, this can be viewed positively – the assessment and feedback have the potential to change behaviour. On the other hand, it warrants caution – participants need to be sensitised to the fact that assessments and feedback only cover a limited time period. With regards to social relationships, there may be periods with less pleasant interactions with a person (e.g., if that person is going through a difficult time), which do not immediately warrant the discontinuation of that relationship. Moreover, difficult social interactions may provide opportunities for growth and be helpful in the long term. What ultimately constitutes adaptive social interaction in daily life is likely highly personal and warrants further investigation. Thus, our feedback prototype should be used with care. Especially, because both the students and focus group participants considered the feedback understandable, but only with the explanations by a researcher. In its current form, the feedback is therefore only suitable for a collaborative discussion between patient and therapist (or researchers) trained for using the prototype, not as a stand-alone tool or in self-help applications.

### Future Research and Implementation in Clinical Practice

Before implementation, further research is needed to determine for which therapeutic approaches and which types of patients the feedback is insightful. Promising candidates may be CBT (Beck & Beck, 2011), IPT (Weissman et al., 2017) and CBAS (McCullough, 2003) which all three use interpersonal behaviours as potential intervention targets.

Furthermore, the participants in our clinical focus group considered the feedback promising – they, however, did not receive feedback on their own data. The next step in the development process will be to test social context feedback for specific clinical cases similar to the Therap-i study (Riese et al., 2021; von Klipstein et al., 2023). Although prior studies already indicate that therapists see value in using ESM and personalised feedback in clinical practice (Bos et al., 2019; Piot et al., 2022; Weermeijer et al., 2023), a specific evaluation of our feedback prototype as well as the assessment procedure combining ESM and PSN from the therapist’s side is warranted.

In the future, the prototype should also be made usable for clinicians and their patients without researcher support. Clinicians applying the prototype likely will require training on how to interpret the figures and use them in practice. Eventually, we aim to achieve implementation in software ready to use for clinicians such as PETRA (Bos et al., 2022), as this reduces therapist burden, and integrates assessment and feedback into existing electronic health records, which is considered important by clinicians (Weermeijer et al., 2023).

The patients in our focus group expressed a clear wish for autonomy over their data, which matches findings from other recent studies (Piot et al., 2022; Weermeijer et al., 2023). Participants have a right to control and access their data under the General Data Protection Regulation (GDPR). In the interest of preventing harm caused by misinterpretation of the data without sufficient expertise, researchers or clinicians may not want to provide the raw data or uncommented feedback. Based on the feedback graphs, patients may make changes to their life, even if the feedback is inaccurate (Leertouwer et al., 2022b). An important safeguard to prevent this could be to provide a commented version of the feedback graphs which includes explanations for interpretation and a summary of conclusions reached during a therapy session (see e.g., von Klipstein et al., 2023).

## Conclusion

Assessing and generating feedback on the social relationships and social interactions of a patient appears promising. Especially, gaining insight into the relationship between social context and psychopathological symptoms may be of value for psychotherapy. We developed a feedback prototype that can provide such insights and serves as a starting point for further clinical research and implementation.

## Footnotes

1 Note that this data collection took place during the Covid-19 pandemic, shortly before the lockdown in December 2021, thus being affected by social distancing measures.

2 The number of social contacts was determined by compiling a list of all unique individuals mentioned as either network member (pre-/post-ESM), interaction partner of a social interaction or both.

3 Figs. 1 and 2 show an anonymized version of a feedback report in which names of network members are exchanged for numbers.

4 Note that the position of the network members is determined by the plot layout in R and has no further meaning, besides connected nodes being pulled closer together. Our code can be found in the supplementary materials on OSF.

5 Note that names of social contacts transferred automatically from the Pre-ESM network and diary assessment into the Post-ESM network assessment, so if names were not included in the collected Post-ESM data anymore, the participant made the conscious decision to remove them.

6 During this data collection, only a few Covid-19-related social distancing measures were still in place. We assumed social life to be more comparable to pre-Covid-19 times.

7 We descriptively examined the differences in the three quantitative ratings (insightfulness, representativeness and understandability) between the two interviewers. There are only minor median and mean differences for understandability and representativeness. There is a slightly larger difference for insightfulness (a difference of 1.5 in the median and 0.9 in the mean on a scale from 1 to 10).
